# Effective Risk Communication for Public Health Emergency: Reflection on the COVID-19 (2019-nCoV) Outbreak in Wuhan, China

**DOI:** 10.3390/healthcare8010064

**Published:** 2020-03-21

**Authors:** Liwei Zhang, Huijie Li, Kelin Chen

**Affiliations:** 1 School of Public Administration, Jilin University, Changchun 130012, China; lihuijie@jlu.edu.cn; 2 Institute of Urban Governance, Shenzhen University, Shenzhen 518060, China; chenkelin@szu.edu.cn

**Keywords:** risk communication, public health, outbreak management, COVID-19

## Abstract

Risk communication is critical to emergency management. The objective of this paper is to illustrate the effective process and attention points of risk communication reflecting on the COVID-19 (2019-nCoV) outbreak in Wuhan, China. We provide the timeline of risk communication progress in Wuhan and use a message-centered approach to identify problems that it entailed. It was found that the delayed decision making of the local government officials and the limited information disclosure should be mainly responsible for the ineffective risk communication. The principles for effective risk communication concerning Wuhan’s outbreak management were also discussed. The whole communication process is suggested to integrate the accessibility and openness of risk information, the timing and frequency of communication, and the strategies dealing with uncertainties. Based on these principles and lessons from Wuhan’s case, this paper employed a simplified Government–Expert–Public risk communication model to illustrate a collaborative network for effective risk communication.

## 1. Introduction

The novel coronavirus (COVID-19; previous official name issued by the World Health Organization is 2019-nCoV, its zoonotic origin is SARS-CoV-2, Severe Acute Respiratory Syndrome Coronavirus 2) occurred in Wuhan, China in December 2019 and January 2020 [1]. This is seen as the third significant outbreak of a coronavirus, following China’s SARS-associated coronavirus (SARS-CoV) that emerged in 2003 and the Middle East Respiratory Syndrome Coronavirus (MERS-CoV) originating in 2012 [1]. Public health outbreaks have deep uncertainty and do not adhere to specific boundaries, which makes risk communication more critical for developing effective public health preparedness strategies [2,3]. An effective risk communication, in general, means that all related risk messages can be presented and shared to participants in a risk communication process openly and timely, aiming to rectify the knowledge gap between the originators of information and those receiving the information, and adjust the public’s behavior to cope with the risk proactively [4,5]. For instance, during the SARS outbreak in China, a perceived lack of information transparency in the initial phase wrecked the effectiveness of risk communication and broadened the impact scope [6]. Time is key to controlling outbreaks. Getting good information and acting on it rapidly can halt outbreaks before they need emergency measures. However, the early history of the COVID outbreak in Wuhan shows information disclosure and delayed decision making, which commonly illustrates an ineffective risk communication associated with COVID-19 [7]. With respect to risk communication for public health emergencies, we reviewed the dynamic process of risk communication of the outbreak management of COVID-19 and used a message-centered approach to identify weaknesses in the risk communication process. We further discuss principles for effective risk communication reflecting on Wuhan’s case. Following these principles and Wuhan’s practice, we will employ a simplified model to illustrate an effective network of risk communication. This study aims to point out an effective process of risk communication based on Wuhan’s case, which can improve the understanding on the cause and impact of the risk and promotes protective behaviors among individuals, communities, and institutions [8]. 

## 2. Method

### 2.1. Data Source

All data in this paper can be divided into three parts: (1) COVID-19 outbreak numbers are retrieved from the National Health Commission of China and the Wuhan Health Commission [9,10]. Some generalized reports and in-depth investigations related to infected cases released by Chinese authoritative media, such as China Business Network, are referred to as well [11]. The purpose of using this data is to reveal how the government manipulate information of actual infected cases. (2) Information about governments’ and experts’ responses and other actions come from Chinese authoritative media and mainstream Internet media including Xinhua News Agency, Caixin News, and Sina News [12,13,14]. The timeline of risk communication of Wuhan’s case is self-summarized from these media sources. (3) To support our case analysis and arguments, interview materials of governmental officials and experts are obtained from Chinese authoritative media like Global Times [15]. 

### 2.2. Message-Centered Approach

The message-centered approach offers scientific and systematic methods to achieve convergence and to avoid asymmetric information around issues of risk. This approach lists best practices designated to building mutually beneficial relationships with risk stakeholders, to helping stakeholders to identify the risk uncertainty and the continuity in communication, and to responding to the communication and informational needs of diverse and changing audiences. Table 1 lists nine aspects of best practices of risk communication on the basis of the message-centered approach.

In sum, this study proceeds as follows: (1) a timeline concerning risk communication of the 2019–2020 Wuhan COVID-19 outbreak is given, (2) the case is then analyzed using the message-centered approach, and (3) a simplified model of effective risk communication is formed in the discussion section.

## 3. Case Description: Outbreak Management of COVID-19 in Wuhan, China 

### 3.1. Background

Wuhan (29°58′ N–31°22′ N, 113°41′ E–115°05′ E) is the capital city of the Hubei province and is the seventh largest city in the People’s Republic of China. As of January 2020, many patients have been found and identified as being infected by a novel coronavirus in Wuhan. Since 12 December 2019, when the Wuhan Municipal Health Commission reported 27 cases of viral pneumonia, including seven critically ill cases, the pneumonia outbreak has received considerable global attention. The COVID-19 cases have covered all provinces in China and have also been reported in other countries, severe areas including Italy, Japan, Republic of Korea, and the US, and all of these cases were exported from Wuhan [16,17]. Chinese scientists have argued that the virus source comes from a seafood market in Wuhan, but a few infected people in Wuhan say they did not visit this seafood market. Pinpointing the actual source of the virus is a main task for current research—different findings have been published [18,19]. An early epidemiological investigation revealed that bats may be the native host of COVID-19 [20]. The available investigations indicate that the virus has the characteristic of human-to-human transmission and is even infectious during incubation. These infections may either be asymptomatic or have symptoms that include fever, cough, and shortness of breath [21,22]. There is no consensus on its basic reproduction number (R_0_ or R-zero), although current scientific studies demonstrate that the range of R_0_ falls within 2.5–6.5 [15,23,24]. There is no complete knowledge on this virus, and epidemiological investigation in ongoing. 

### 3.2. Risk Communication Timeline of COVID-19 Outbreak Management

Risk communication is a combination of two aspects, namely, internal communication and external communication. Internal communication refers to a situation where risk assessors and managers develop a common understanding of their tasks and responsibilities. It enables risk assessors and managers to appraise the potential impact and all possible outcomes based on the available information. Meanwhile, external communication enhances stakeholders’ awareness of the negative impact of the risk and their recognition about their roles in risk governance and initiation of different behaviors [25,26]. Following this distinction and referring to China’s regime, we define internal communication as the communicative process among governments and the academic community, because the majority of academic institutions are affiliated to the government or funded by officials. Furthermore, we posit that external communication is related to the information sharing between the government and the public. In accordance with the development of the COVID-19 outbreak, Table 2 illustrates the processes of internal and external communication with other events related to COVID-19 information. The period is from the initial report to Wuhan’s lockdown.

## 4. Analysis on Risk Communication in COVID-19 Management: A Message-Centered Approach

From the timeline of risk communication in Wuhan, we can generalize three aspects related to risk communication: government’s decision making, government’s information dissemination, and risk interpretation. Following nine aspects in the message-centered approach, we selected four aspects relevant to three aspects of COVID-19 risk communication to analyze this example of ineffective risk communication. 

### 4.1. Infuse Risk Communication into Policy Decisions

At the beginning of the outbreak, the Wuhan government did not infuse a scientific risk communication into decision making and regarded the outbreak as a common public health issue instead of an emergency without a precise investigation and consensus about the epidemiological characteristics of COVID-19. A comment published by the New York Times speculates that the decision making was based on social stability [27]. Therefore, there was no adequate preparedness for outbreak management, including a timely warning to the public and active countermeasures for the risk. Organizational characteristics should be taken into consideration in the analysis of risk communication. Organizations often have a vested interest in a particular interpretation of risk [28,29]. The occurrence of the outbreak corresponded with China’s political season, when officials gather for annual meetings of the People’s Congresses—the Chinese Communist Party (CCP)-run legislatures for discussing policies and praising government. Bad news is inappropriate at this time. As the governor of Hubei, Wang Xiaodong, pointed out, “political issues are at any time the most fundamental major issues” [27]. At the same time, the harmonious and happy atmosphere brought about by the Chinese Lunar Year Festival in society foreboded the start of the most massive annual population migration, which is called the “Tide of Going Home”. If the outbreak information were to spread, it would influence migration and intensify social fear. Based on balancing various considerations, the government’s decision making had to depend upon maintaining political image and social stability, because the outbreak management might disturb the social order. In an interview, Zeng Guang, the chief scientist of epidemic of Chinese Center for Disease Control and Prevention, indirectly admitted that the main problem came from the local government’s decision making. Zeng said that the government is prone to balance many factors in decision making, such as politics, stability, and economy, and expert opinion is often partly considered by the government [15]. In addition, a prediction of the epidemics trend of COVID-19 in China conducted by Dr. Zhong Nanshan’s group indicates if government control was applied five days earlier, the epidemic would have been effectively suppressed [30]. We can infer from this research that the government’s reactive action should have been adopted earlier.

### 4.2. Present Risk Messages with Honesty

**Figure 1:**
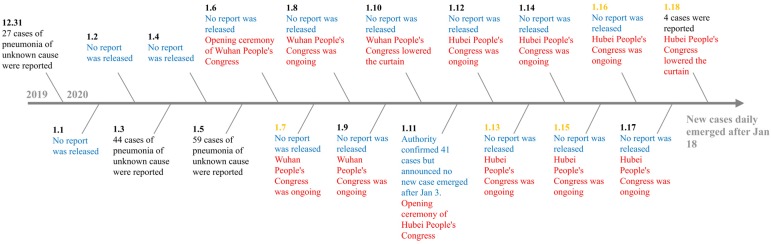
Timeline of official reports about infected cases by the Wuhan Health Commission. **Source:** This timeline is a processing of data from Website of Wuhan Health Commission and a generalized report and in-depth investigation from China Business Network [10,11]. Note: Blue represents “No report”; Red represents Wuhan and Hubei’s Congress; Date in yellow represents confirmed infected case of medical worker.

Designing open, accurate, and consistent messages is a vital premise for communication preparedness [31] (p. 105). In the management of COVID-19, the whole external communication reflects the possibility that the government concealed information about the outbreak. During the political congress, officials did not report new cases and repeatedly stated that no medical workers were infected, but the reality was that new cases were being diagnosed every day [11]. Interestingly, officials reported new cases after the congress lowered the curtain. Figure 1 shows a timeline of official reports about infected cases by the Wuhan Health Commission. 

Besides, the way that the government handled the information of the outbreak made the information more ambiguous. After the initial outbreak of COVID-19, conspiracy theories and rumors spread online regarding the origin and scale of the virus. Various social media posts claimed that the virus was a bio-weapon or an American conspiracy aimed at containing China [32]. The government was busy refuting these rumors. The Chinese Medical Doctor Association makes a daily statistic of rumors on social media. From 18 January to 10 March, the government has refuted 434 rumors covering virus source, preventive measures, disease impact, research achievements, etc. [33]. From a theoretical perspective, such an information chaos may impair the government’s credibility. Trust is one major objective in risk communication. The timely disclosure of relevant information has a positive impact on building trust. On the contrary, stalling or delaying reporting will undermine the trust in risk communication and governance [34] (pp. 214–215).

### 4.3. Account for the Uncertainty Inherent in Risk

The literature of risk communication indicates that risk combines the known unknown and the unknown unknown, remaining equivocal in risk messages means acknowledging that uncertainty exists and framing messages within that inherent uncertainty, for example, “We do not yet have all the facts” and “Our understanding of these factors is always improving” can be used to preface risk messages [8,35]. Incipient risk external communication used a series of certain expressions, which conveyed the wrong perception of COVID-19 to the public. The actions of the authorities in attacking whistleblowers who privately delivered the clinical characteristics of the coronavirus on social media further enhanced people’s risk perception about the outbreak. The public tended to believe that the disease had no characteristic of human-to-human transmission. As the epidemiological investigation went deeper, new conclusions made the authorities change their statement, using expressions such as “no evidence shows the disease has the characteristic of human-to-human transmission”. However, the public could not realize the characteristic of human-to-human transmission from such an expression, and thus had no perception for self-prevention. On 20 January, Dr. Zhong Nanshan confirmed the epidemiological characteristic of human-to-human transmission, which was a sudden turn in the content of risk communication. Therefore, the risk communication was not evidence-based but arbitrary, because it failed to conform to a dynamic process as the understanding of the risk evolved.

### 4.4. Acknowledge Diverse Levels of Risk Tolerance

People have widely varying capacities to process risk messages, including scientific and technical understandings of risk. Diverse levels of risk tolerance or perception amongst different people are due to ambiguity in public health issues, multiple sources of information, and various communication behaviors [36] (pp. 224–265). In the risk communication of COVID-19, some cases have shown that experts or the government failed to utilize understandable explanations about the epidemiological characteristics of the outbreak, the scientific principles in the prevention measures, and the curative effect of therapeutic activity. The public remained ignorant or fearful. Many people used extreme methods for self-protection. Under the media’s misleading influence, lots of people snapped up medicines that have not been proven to have a curative effect or hoarded masks and respirators. A research conclusion cited by Xinhua News Agency indicates that a preliminary study shows that Chinese patent medicine *Shuanghuanglian* can inhibit the novel coronavirus. The consequence was that lots of people queued up purchasing *Shuanghuanglian* at midnight. An expert clarified that this study is not a detailed research but only preliminary, and indicated that scientific expression should not be exaggerated [37]. In addition, many people had no perception of the outbreak. Some extremists decided against self-protection and even vandalized public facilities that are used to community quarantine.

## 5. Principles for Effective Risk Communication: Lessons from Wuhan

The lessons from the outbreak management of COVID-19 in Wuhan stress the need for effective risk communication to prepare in advance for an infectious disease emergency. We can consider the following principles:

### 5.1. Accessibility and Openness of Risk Information

Accessibility and openness enhance the public’s perception that they are fully informed about risk and that they are partners in sharing the risk. Risk communication must consist of an interactive process where all parties are given access to multiple messages representing all relevant views. Identifying the points of convergence serves as a means for making sense of these interacting arguments, which leads to forming a consensus on the uncertain issue [8] (p. 17). Wuhan’s case reflects the prerequisite of a message-centered approach, which means that the institution can be a barrier to information disclosure. The government’s monopoly of information harms the public’s right to know, and the fact that politics is the most important issue restricts the voice of the academic community. Reluctant information disclosure hampered the public’s self-protection and exasperated negative impacts of the outbreak. As a consequence, the tardy strategy of lockdown broadened the outbreak scope subjectively due to people’s migration. Meanwhile, in accordance with the characteristic of COVID-19, namely, that it can transmit in incubation, the public’s ignorance of self-protection meant that the disease was able to infect others during gatherings and contact between people in an unperceivable way. Risk communication strategies require information sharing and establishing networks of working relationships among individuals, groups, and agencies. Establishing these relationships necessitates the accessibility and openness of information, which is the premise of collective action.

### 5.2. Communicate Early and Often About Risk

Risk communication should begin as soon as a risk has been identified and should continue as new information becomes available [38] (pp. 72–73). For an unknown disease, communication should avoid using certain conclusions or expressions when clinical and epidemiological investigations are ongoing. Once an updated investigation is available, the information ought to be disclosed immediately. Any delay will likely lead to unexpected consequences. The government of Wuhan’s first step to respond to the unknown disease was not to initiate a comprehensive investigation, but to silence and punish “rumormongers”. The reality proves that “rumormongers” are innocent and that the “rumor” indeed functioned as an early warning. On 7 February, Dr. Li died from COVID-19, and his death unleashed an upsurge of emotion, with the public strongly criticizing the government’s management of their response to the initial outbreak [39]. The shocking nature of risk can sometimes paralyze an organization, and even a holistic governance network. Therefore, inadequate preparedness, such as the shortage of goods, loose management in communities, and disordered collaboration amongst agencies, put the Wuhan and Hubei governments into a passive situation and undermined their institutional trust. As a result, the perception was that the organizations were immorally withholding risk information from the public. Effective risk communicators should make immediate contact with the public about risk and maintain regular information to the public concerning risk levels and tendency throughout an incident. 

### 5.3. Strategic Method for Communicating Uncertainty

Risk communication often contains uncertainty information associated with technologies, behaviors, medical procedures, etc. To be effective, such messages need to incorporate ideas, images, and logic that will promote comprehension among the lay public [40] (p. 213). Due to the knowledge gap, experts and laypeople tend to perceive risks in different ways and tend to use different terms to discuss them [41]. Communicating uncertainty effectively requires assessing the different levels of perception among different audiences, and utilizing an evidence-based approach to convey uncertainty. As a tool for communication, the evidence-based approach has been used in quantifying and delivering uncertainty. In risk communication, particularly in public health, using equivocal expressions is most effective when they avoid overly certain predictions [8] (p. 23). However, equivocal expressions are subjective, because of the heterogeneity of different people’s understandings. Thus, evidence-based communication aims to translate the verbal probability, such as “possible”, “probably”, and “maybe”, into numerical probability, which can convey the degree of uncertainty in an unambiguous way [42].

Accurate dissemination to and among separate groups requires specialized communication strategies [43] (pp. 28–29). Especially for uneducated persons, using vivid or graphic metaphors for exemplification can illustrate the epidemiological characteristics in a direct, simple, and visual way, which can match people’s various capacities to understand the transmission and R_0_ of the epidemic. The outbreak management of COVID-19 has testified that the public cannot form a general perception of risk if the strategic communication is absent. For instance, many people fail to realize and comprehend epidemiological characteristics, leading to a lack in people’s protection and ignorance toward management policies. Decision making in risk governance is not based on the technical aspects of the risk alone. Audience perceptions and concerns must be considered if risk decisions, and their communication, are to be successful [34] (p. 147). Risk communication is not only designed to deliver the knowledge, but also to change the public’s attitude in order to make the public accept the general arrangement of outbreak management.

## 6. Discussion: A Simplified Model of Government–Expert–Public Risk Communication

**Figure 2:**
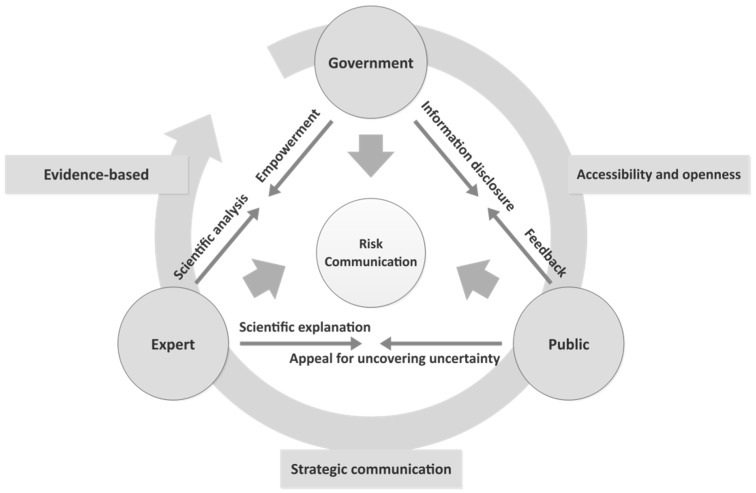
Government–Expert–Public Risk Communication model.

Following the message-centered approach, we put forward three principles for effective risk communication for a public health emergency. Generalizing these principles, three main actors can be abstracted, namely, the government, experts, and the public. The risk communication network reflecting Wuhan’s outbreak management consists of these three actors. In order to simplify their complex communications, we employed a model to demonstrate the communicative interaction that will enable a better understanding of the communication strategy and principles. The model is presented in Figure 2 below.

In this model, the three components of communicative interaction are government–public, government–expert, and expert–public. The government is the core decision maker in the risk governance process, and all of the government’s behaviors will have a profound impact on the effectiveness of governance. 

For government–public communication, which is a typical external communication, the government’s responsibility is to convey adequate and accurate information to the public, which means that information disclosure is accessible and open. Government officials are often frustrated by what they perceive to be inaccurate public perceptions of risk and unrealistic demands by the public for risk reduction [43] (p. 1). In addition, this communication results in a response from the receiver, which can then be evaluated against the desired response [44]. The public’s feedback enables the government to adjust its emphasis of information delivery and provide information in relation to the public’s own interests and values—communication can be most effective when it reflects an understanding of what the public wants to know [43,45]. The existing literature indicates that a challenge identified in implementing openness in risk communication is that it is difficult to decide what to present and what not to present. For instance, complete transparency causes possibly unjustified fear among members of the public [46]. As we mentioned above, the initial intention behind the Wuhan government concealing information was based on the consideration of maintaining social stability. Therefore, transparent risk communication is just as necessary, but is simultaneously difficult to achieve in practice due to many complex decision situations. Information disclosure is a technical measure that needs to balance different factors such as public risk tolerance and possible subsequent outcomes. 

Government–expert communication is a primary element of risk assessment and decision making, which can be seen as internal communication. Risk is related to professional knowledge and technology. The very essence of responsible and rational action is to make viable and morally justified decisions in the face of uncertainty based on a range of expert judgments and assessments. At the stage of risk assessment, the consensus of experts’ judgments about the risk will ensure to assign an accurate probability for each possible consequence, to initiate each action, and to establish the rational decision as that which minimizes the negative outcomes and maximizes the expected benefits [34,47,48]. Governments ought to empower experts to work on comprehensive and detailed research on the uncertainty of a risk issue rather than restrict experts’ voices out of other considerations, including politics or self-interest. The academic community should fully focus on the scientific analysis of the risk and sharing findings, data, and materials. In modern society, risk is an interdisciplinary issue. For public health risk, specialists in life and social sciences, biomedicine, and public health must seek better answers from multiple disciplines. Consensus on evidence-based analysis is the foundation of decision making. Uncertainty due to disagreement (experts, opinions, language) must be analyzed and disclosed, which aims to avoid misleading by ambiguous and diverse information [49].

Expert–public communication is dedicated to bridging the gap between expert and public views on public health issues through strategic communication. It represents external communication. A great challenge of risk communication is not only to convey knowledge, but also to find ways to convey comprehensive information that reflects uncertainty and empowers the public to make fact-based decisions about health [50]. The public always fails to understand the complex professional knowledge about the risk, so experts’ responsibility is to translate the professional knowledge into simple and explicit content which can be easily understood. In Wuhan’s case, a doctor named Zhang Wenhong said frankly, “You are unable to understand what I am saying definitely, because we read different books. You know every word in my sentence, but you do not know what I mean”. Then, Dr. Zhang used quite straightforward language to explain how Shanghai is coping with the outbreak by integrating resources. His explanation received the public’s praise and support [51]. The public need to express their appeals of uncovering uncertainty to experts when they encounter their unknown knowledge about the risk rather than seeking some unproven information or even rumors. 

Risk communication responsibility should be a balance between being neither too centralized nor too decentralized [35] (p. 112). Different actors must fulfill their responsibility according to their roles and keep the communication network running. Accordingly, this model underscores the importance of partnerships in risk communication. Within the communication network, the government should and must play a leading role in communicating risk, and the proposed model offers a guideline to achieve this goal. Based on Wuhan’s case, the government will continue to face challenges as uncertain and unexpected outcomes occur. Therefore, the government must collaborate with other actors to share information in a timely and effective manner, which enables every actor to adopt preparedness in advance for tackling any unexpected incident in the future.

## 7. Conclusions

This article focused on how ineffective risk communication impeded the emergency response in Wuhan’s outbreak management and discussed principles for effective risk communication. After the timeline of risk communication progress in Wuhan was given, we tried to illustrate the effective process and attention points of risk communication reflected from Wuhan’s case. Following the message-centered approach, it was found that the Wuhan government did not infuse a scientific risk communication into policy decision, the local government stalled reporting and handled the information publicity in an ambiguous way which undermined public perception associated with COVID-19, and the authorities failed to treat it with the inherent uncertainty and different levels of risk perception of COVID-19, which worsened the circulation of rumors and led to public panic to some extent. The lessons from the outbreak management in Wuhan suggested that the accessibility and openness of information should be enhanced to form convergent points in the whole communication process, especially when dealing with uncertain issues, should keep the public regularly and timely informed, and should take care of the communication strategies dealing with uncertainties. Then, a simplified model of risk communication was employed to illustrate a collaborative network for effective risk communication. The government, experts, and the public should be involved in time, contributing diverse views and fulfilling respective responsibilities. In China’s case, the government normally plays a leading role but sharing information in a timely and effective way needs to be solved in practice in the long run.

## Figures and Tables

**Table 1:** The message-centered approach and its best practices of risk communication.

Best Practices for Risk Communication	Description
Infuse risk communication into policy decisions	Policies about risk may evolve and be communicated in a variety of ways. Decision making needs to be based on constant risk communication.
Treat risk communication as a process	Effective risk communication is a dynamic, interactive, and adaptive process.
Account for the uncertainty inherent in risk	Using equivocal messages to convey risk information.
Design risk messages to be culturally sensitive	Risk communication should fit specific features of the audience. These features include gender, education, age, and culture.
Acknowledge diverse levels of risk tolerance	People have widely varying capacities to process risk messages, including scientific and technical understandings of risk.
Involve the public in dialogue about risk	Risk communication dialogues should involve collaborations between the government, industry, and citizens that are open, inclusive, and deliberative.
Present risk messages with honesty	Risk communication should be an open, honest, and frank process, instead of essentially manipulative.
Meet risk perception needs by remaining open and accessible to the public	Honest communication is accessible and open as well, which means that the public can receive messages by various channels.
Collaborate and coordinate about risk with credible information sources	Coordination of risk communication strategies requires information sharing and establishing networks of working relationships between groups and agencies.

**Source:** This table is a content summary from Sellnow et al. [8] (pp. 19–29).

**Table 2:** Risk communication timeline of COVID-19 outbreak management.

Date	Internal Communication	External Communication	Other Events Related to COVID-19 Information Disclosure
27 December 2019	Initial report: A doctor named Zhang Jixian reported cases related to COVID-19 to the Health Commissions of Wuhan and Hubei Province.		
29 to 30 December 2019	Initial investigation: The Health Commission of Wuhan required that all medical institutions must investigate patients who have such unknown pneumonia privately.		Private whistleblowing: An ophthalmologist named Li Wenliang used social media to whistleblow that seven cases associated with SARS were identified and that detailed experimentation was ongoing.
31 December 2019	Action at the Central level: The National Health Commission’s initial action for organizing a group to investigate the outbreak.	Initial announcement: The Health Commission of Wuhan publicly announced the outbreak but highlighted that there was no evidence of human-to-human transmission, that no medical workers were infected, and that this outbreak could be prevented and controlled.	
1 January 2020			“Attacking the rumor”: Li Wenliang and a further seven doctors were interrogated for “spreading rumors”.
3 to 5 January 2020	Further Evidence submitted: An academic group from Fudan University, Shanghai, found the SARS-like coronavirus and submitted the finding to the National Health Commission on 5 January.	Official announcement: Official announcement repeatedly conveyed that there was no evidence of human-to-human transmission, that no medical workers were infected, and that this outbreak was preventable and controllable.	
6 January 2020		Response at the Central level: The Chinese Center for Disease Control and Prevention (CCDC) activated II Level Response of Public Health Emergency.	
7 January 2020	Supreme direction: The Leader of the Chinese Communist Party (CCP), Xi Jinping, arranged countermeasures to respond to this outbreak.	Official explanation on confirming a new coronavirus: Chinese official media announced there is a new coronavirus that has emerged in Wuhan.	
10 January 2020		Scientific explanation and risk assessment from expert: A medical expert at Peking University indicated that the outbreak can be controlled.	
15 January 2020		Upgrading the response at the Central level: CCDC activated the highest I Level Response of Public Health Emergency.	
16 January 2020		Official risk assessment: Wuhan municipal authority informed that there is low risk in persistent person-to-person transmission.	
17 January 2020	Divergence in the epidemiological investigation: An academic group from Hong Kong University found evidence of person-to-person transmission and infection without symptoms. An expert named Kwok-Yung Yeun reported the finding to CCDC.		
20 January 2020	Supreme command: CCP’s leader, Xi, required local governments to highly focus on controlling the outbreak by applying proper measures.	Verified epidemiological characteristics of COVID-19 from the academic community: A famous respiratory physician, Zhong Nanshan, confirmed the evidence of person-to-person transmission.	
22 January 2020		Response at the provincial level: Hubei Province activated II Level Response for Emergency.	
23 January 2020	The Wuhan government officially announced that the whole city would be under large-scale quarantine at 10 a.m., which meant the beginning of formal and comprehensive management on this outbreak.		

**Source:** This timeline is a summary of paraphrased reports from China’s official media.
